# Removing an Embolized Peripheral Intravenous Catheter from the Left Caudal Lung Lobe of a Dog via Intercostal Thoracotomy

**DOI:** 10.3390/vetsci13010017

**Published:** 2025-12-24

**Authors:** Samantha Masca, Margaret Goodale, Anke Langenbach

**Affiliations:** Veterinary Surgical Centers (VSC), Vienna, VA 22180, USAmeggoodale@gmail.com (M.G.)

**Keywords:** intravenous catheter embolism, thoracotomy, dog

## Abstract

An 8-month-old Standard Poodle was referred to a surgical specialist for suspected peripheral intravenous catheter (PIVC) embolism originating from the right cephalic vein. Upon removing the 20-gauge PIVC, the tip appeared to be 2 cm shorter. Radiographs were obtained for the localization of the PIVC embolism, but no abnormalities were seen. A computed tomography scan of the whole body was performed. PIVC embolism was observed due to a difference in Hounsfield units between the catheter tip and the surrounding lung parenchyma in the ventral aspect of the left caudal lung lobe. A left lateral thoracotomy in the seventh intercostal space was then performed, leading to the successful retrieval of the catheter tip while allowing the lung lobe to be spared. This case report describes a PIVC embolism migrating to the lung parenchyma and its successful surgical removal.

## Simple Summary

An 8-month-old Standard Poodle presented with transection of a peripheral IV catheter tip upon removal from the antebrachium. The foreign material was not seen on radiographs. A computed tomography scan was used for localization, and the IV catheter tip was found in the ventral aspect of the left caudal lung lobe. Surgery was successfully performed to remove the foreign material from the lung parenchyma. In the event of IV catheter embolism to the lung, it is possible to spare the lung with surgical removal.

## 1. Introduction

Peripheral intravenous catheterization (PIVC) is a commonly utilized procedure in veterinary medicine for the administration of a wide variety of treatments, including fluids, medications, and blood products [1]. Reported complications associated with PIVC range from 12.1 to 43% according to a variety of studies [2,3,4,5], including phlebitis, extravasation [1,2,5], dislodgement or occlusion, and embolism [1].

PIVC embolism is a rare, potentially fatal complication where a piece of the intravenous portion of the peripheral catheter breaks off and travels intravenously to the heart or lungs with a chance to cause a pulmonary embolism [1]. When PIVC embolism occurs, diagnosis usually comprises multiple imaging modalities, including radiographs, ultrasound, computed tomography (CT), or even fluoroscopy [6,7,8]. Reported clinical signs range from asymptomatic to life-threatening. Treatment aims to identify the broken piece and remove it prior to the development of further complications. In humans, clinical signs range from asymptomatic to arrhythmias, respiratory symptoms, such as cough, dyspnea, thoracic or pleural pain, and sepsis [6,9] with migration to peripheral veins and pulmonary arteries, as well as the right atrium and right ventricle. PIVC foreign bodies and potential embolisms are less well-described in veterinary medicine. A total of seven patients with PIVC embolism in the peripheral vein or the pulmonary artery have been described [6,8,10].

Intervention is an important component when PIVC embolism occurs. Several case reports have described the diagnostic and treatment approaches in human medicine, which include minimally invasive procedures such as percutaneous endovascular retrieval [6] and open surgery procedures such as a venotomy [11] or a thoracotomy approach for the removal of a PIVC embolism [12]. To the authors’ knowledge, this is the first case report to describe the utility of CT for confirming PIVC embolism into the lung parenchyma and the use of a left lateral intercostal thoracotomy approach to remove the migrated PIVC located in the ventral aspect of the left caudal lung lobe of a dog.

## 2. Detailed Case Description

**Figure 1:**
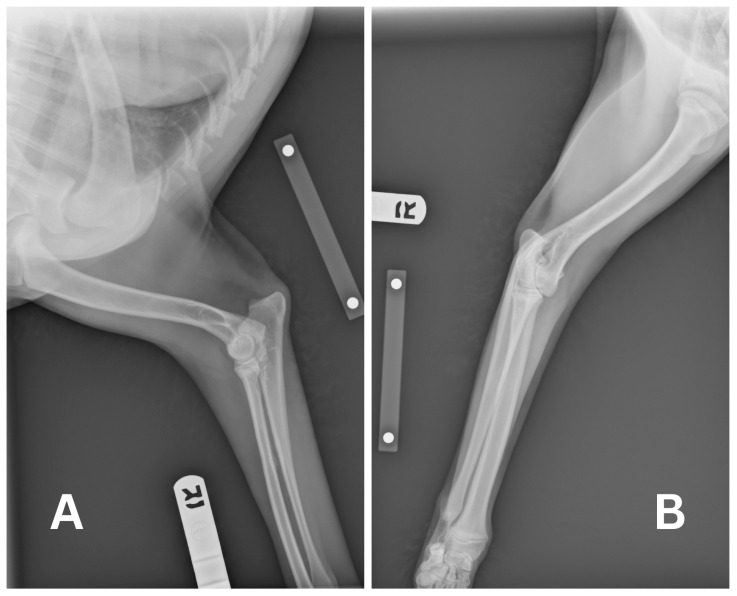
Radiographs of the right thoracic extremity in lateral position (**A**) and posterior–anterior position (**B**) were taken by the referral veterinarian to observe the embolized intravenous catheter. No remnants could be seen.

An 8-month-old, 18.7 kg, female intact Standard Poodle presented to her primary care veterinarian for dental surgery to extract the tooth root of 404. The tooth was chipped during a play date with another dog. She had previously been healthy with a bout of kennel cough a month prior and left-sided conjunctivitis, which had resolved. Her pre-operative blood work was within normal limits, other than mild lymphocytosis (lymphocytes 5412/μL) and monocytosis (monocytes 1476/μL). Her coagulation panels were checked and found to be within normal limits. She was premedicated with 0.02 mg/kg of acepromazine (10 mg/mL, PromAce, Boehringer Ingelheim Animal Health, Duluth, GA, USA) and 0.5 mg/kg of morphine (10 mg/mL, Morphine Sulfate, Pfizer, New York, NY, USA) intramuscularly. Additionally, 1 mg/kg of maropitant (Cerenia, Zoetis, Parsippany, NJ, USA) was administered IV after a right cephalic 20-gauge catheter was placed. General anesthesia was induced with propofol (10 mg/mL, PropoFlo 28, Zoetis, Parsippany, NJ, USA) given to effect IV, for a total of 7.7 mL, and maintained on isoflurane (Isospire, Dechra, Northwich, UK) in 100% oxygen through a cuffed endotracheal tube. Dental radiographs showed that there was no retained tooth root, and the alveolar cavity was closed with simple interrupted sutures using 4–0 Monocryl. The patient recovered smoothly and was discharged with 2.0 mg/kg of carprofen (Rimadyl, Pfizer, New York, NY, USA) and 16.0 mg/kg of clindamycin (Clintabs, Virbac, Westlake, TX, USA), both to be taken orally every 12 h. Upon removing the 20-gauge PIVC prior to discharge, the tip was accidentally cut horizontally from the catheter hub with bandage scissors. Two-view radiographs of her right thoracic limb were taken immediately but no visualization of the suspected remnant could be identified (Figure 1). She was promptly referred for further work-up.

**Figure 2:**
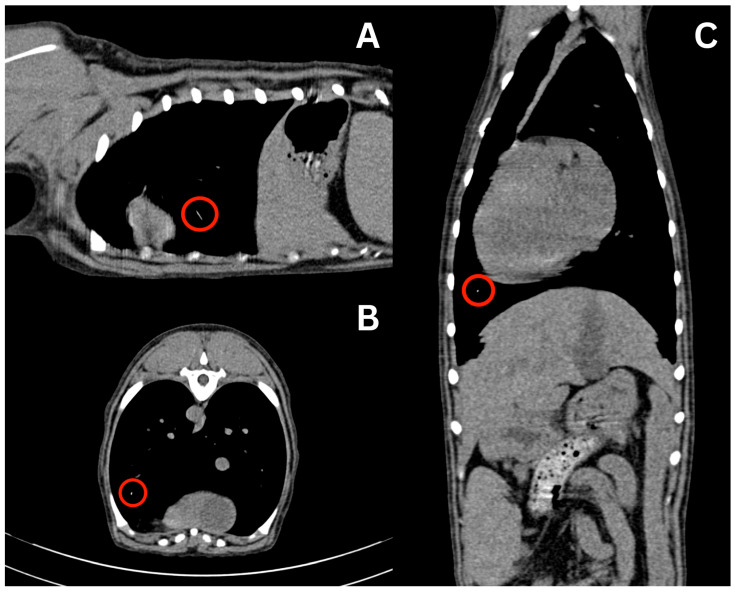
CT scan displaying hyperattenuating linear structure in the left ventral lung lobe, suspected at the time to be the migrated intravenous catheter estimated to be 20 mm in length. Sagittal (**A**), axial (**B**), and coronal (**C**) views are displayed with visualization of the suspected structure in each view indicated by the red circle.

At the time of presentation to the emergency department, the patient had no clinical signs. Physical examination was unremarkable other than her missing 404 tooth with stitches in place and a shaved right cephalic region from the prior PIVC site. Remnants of the PIVC were compared to an actual 20-gauge PIVC. The estimated missing catheter tip was 18–22 mm long. At this point, a full-body CT scan was recommended, for which the patient was anesthetized on the same day. The patient was premedicated with 0.004 mg/kg of fentanyl (50 mcg/mL, Fentanyl Citrate, Hospira, Lake Forest, IL, USA) IV, and then induced with 0.2 mg/kg of midazolam (5 mg/mL, Midazolam Injection, Avet Pharmaceuticals, East Brunswick, NJ, USA) IV and propofol to with a total volume of 6 mL IV. Anesthesia was maintained with isoflurane in 100% oxygen. The patient was placed in sternal recumbency for the CT scan (Somatom Emotion 16, Siemens Healthineers, Malvern, PA, USA). Protocol parameters were as follows: kVp: 130; exposure time: 600; and slice thickness: 0.75 mm. Initially, the full-body CT scan was unremarkable, with no linear hyperattenuating structure visualized in the cephalic vasculature, pulmonary vasculature, or cardiac silhouette. The skull, cervical region, thorax, abdomen, pelvis, and pelvic limbs were reported as normal, and no remnant of the PIVC could initially be identified. No complications occurred during anesthesia and recovery post-operatively was uneventful. The patient was discharged the same day. Since the PIVC had to be present in the patient, a misdiagnosis was suspected. The authors requested a second opinion from another board-certified radiologist, and the CT report was amended as a small linear hyperattenuating structure was visualized in the ventral aspect of the left caudal lung lobe, as seen in Figure 2. It was assumed that this was the PIVC tip due to the difference in Hounsfield Units (HU) between the linear structure of the PIVC (60 to 100 HU) compared to the surrounding blood vessels (30 to 60 HU) and lung parenchyma. The surrounding pulmonary parenchyma was normal with no pulmonary thromboembolism.

Due to these findings, the patient was readmitted the next day for a left lateral thoracotomy with potential lung lobectomy. A repeat CT scan was recommended to verify the location of the PIVC tip immediately prior to surgery. A complete blood count (CBC) and non-steroidal anti-inflammatory drug (NSAID) panel, consisting of alkaline phosphatase, alanine aminotransferase, aspartate aminotransferase, blood urea nitrogen, and creatinine performed prior to repeat CT imaging, showed no abnormalities except eosinopenia (0.00 K/uL; reference range: 0.06–1.23 K/uL). The initial partial thromboplastin time (PTT) result (>300.0 s with a reference range of 72–102 s) was considered erroneous; repeat testing confirmed a value within the reference range of 93.0 s. The patient underwent general anesthesia and was premedicated with 1 mg/kg of maropitant and 0.19 mg/kg of hydromorphone (10 mg/mL, Hydromorphone Hydrochloride, Pfizer, New York, NY, USA) IV. She was then induced with 0.2 mg/kg of midazolam IV and propofol to affect with a total of 6 mL IV (3.2 mg/kg total). She was maintained on isoflurane in 100% oxygen with IV crystalloid fluids at 5 mL/kg/h. CT verified that the linear hyperattenuating structure remained in the peripheral pulmonary artery within the ventral aspect of the left caudal lung lobe.

**Figure 3:**
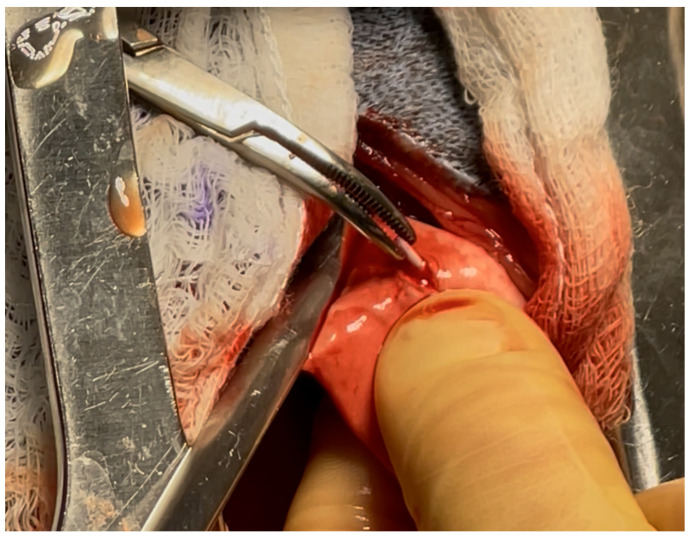
Intraoperative incision into the left caudal lung lobe over the embolized PIVC fragment and surgical extraction of said fragment with hemostats. The lung was occluded proximal to the embolized PIVC with atraumatic straight Doyen forceps.

**Figure 4:**
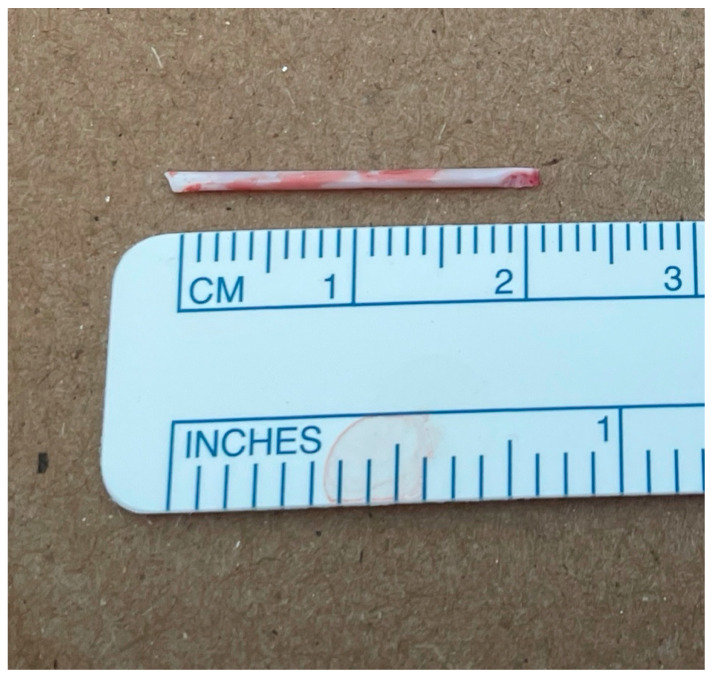
Intravenous catheter tip of 20 mm in length was retrieved from the left lateral lung lobe via lateral thoracotomy.

The patient was transferred to the operating room, placed in right lateral recumbency, aseptically prepped, and draped. A dose of 770 mg Cefazolin (Cefazolin Sodium, West-Ward Pharmaceuticals, Berkeley Heights, NJ, USA) was administered IV as prophylactic antibiotic. The patient was provided a 100 mg dose of ketamine (Ketamine Hydrochloride, Dechra, Northwich, UK) intra-operatively for further analgesia. A standard left intercostal thoracotomy was performed at the level of the seventh intercostal space. Upon entering the chest cavity, the left cranial and left caudal lung lobes appeared within normal limits on visual inspection. Foreign material was palpated on the ventral aspect of the left caudal lung lobe. Initially, the foreign material was digitally secured in what was presumed to be a small artery in the left caudal distal lung lobe. Doyen atraumatic intestinal forceps were used to clamp the dorsal area to the foreign material to hold the bronchus/artery/veins in a closed position to avoid any movement of the catheter tip. A #15 blade was used to incise a 2 mm opening at the level of the foreign material, as shown in Figure 3. The 2 cm long PIVC tip of was removed, as shown in (Figure 4). The lung incision was closed with 5-0 polydioxanone in a simple interrupted pattern. No other abnormalities were found. The thorax was lavaged with saline and the left caudal lung lobe was submerged in saline and leak-tested at 20 cm H_2_O pressure; no leaks were identified. A 12 Fr MILA chest tube was placed dorsocaudally to the skin incision and secured with 3-0 polyamide in a simple interrupted pattern. Circumcostal sutures were placed in a simple interrupted pattern with 0 polydioxanone. Muscles were opposed and closed in several layers in a simple continuous pattern with 2-0 polydioxanone. The subcutaneous tissue was closed using 2-0 polydioxanone in a simple continuous pattern, and the skin was closed using 3-0 poliglecaprone in an intradermal pattern. A total of 7 mL (13.3 mg/mL) of bupivacaine liposome suspension (Nocita, Elanco, Indianapolis, IN, USA) was injected along the incision at the time of closure. The patient recovered uneventfully from anesthesia. She stayed overnight in the hospital for observation, and no complications were seen. The chest tube was removed the next day prior to discharge into the owner’s care. The patient was prescribed gabapentin (Gabapentin, Strides Pharma, Bengaluru, India) 10 mg/kg PO q8–12 h as needed for pain control, tramadol (Tramadol Hydrochloride, Amneal Pharmaceuticals, Bridgewater, NJ, USA) 4 mg/kg PO q8–12 h as needed for pain control, carprofen 2.2 mg/kg PO q12 h for 7 days for anti-inflammatory effects, and trazodone (Trazodone Hydrochloride, Torrent Pharmaceuticals, Ahmedabad, India) 2.7–5.3 mg/kg PO q8–12 h for sedation during recovery. Follow-up was conducted via telephone, and the patient was reported to be normal without any respiratory compromise at 2 weeks post-surgery.

## 3. Discussion

To the best of the authors’ knowledge, this is the first reported case of surgical removal through the lateral thoracotomy of a PIVC fragment in the pulmonary parenchyma while sparing the affected lung lobe. PIVC foreign bodies are rare in veterinary medicine and are more commonly seen in humans due to the wider use of interventional radiological procedures. Successful retrieval has been documented for people undergoing removal, ideally through a percutaneous intravascular procedure, but open procedures have also been performed depending on the location of the catheter fragment. Common fragment locations in human medicine are the right ventricular chamber and the pulmonary artery [12,13]. With regard to this case, we suspect that the PIVC fragment migrated to the left lung lobe through the right cephalic vein into the main pulmonary artery and diverged into the left pulmonary artery. The explanation of how the foreign material ended up in the left lung lobe is multifactorial, including the size, shape, and momentum of the foreign body at the time. One theory is that the patient’s position at the time of catheter transection may have had an effect. The patient was lying in left lateral recumbency for a short amount of time after the PIVC was transected. This could have led to increased cardiac output into the left pulmonary artery over the right pulmonary artery, lodging the PIVC in the left lung lobe [14].

In this case, the removal resulted in resolution, thereby avoiding the risks of potential complications from migrated PIVC, such as heart chamber penetration, pneumothorax, sepsis, and thromboembolic disease, among others, that could have occurred if the PIVC was left in the patient. Risks and benefits must be weighed, although the overall standard of care in human medicine is fragment localization with CT and the removal of the foreign plastic fragment [6,13,15]. Such general recommendations are missing in veterinary medicine as these cases are rare. A case series by Culp et al. involved five dogs using percutaneous endovascular retrieval with excellent results [6]. In the current study, an open approach was chosen due to the likelihood of not being able to reach this PIVC embolism deep in the parenchyma of the lung. An open removal with PIVC embolism is an uncommon occurrence as most reported cases involve venous system retrieval, including embolism to the cephalic vein, omobrachial vein, right pulmonary artery, left pulmonary artery, and right main pulmonary artery, as described by Culp et al. [6]. There are no published cases of PIVC migration into the lung parenchyma in veterinary medicine.

Very few cases have reported the use CT of scans for the identification of foreign materials, as the materials are commonly localized with radiographs [6]. However, a case study described by Frau et al. utilized CT in two cases of PIVC embolization when radiographs and ultrasound failed to identify the foreign material. A CT scan of the thoracic limbs localized the foreign material where it was trapped due to early torniquet application [7]. Although radiography remains the mainstay for the localization of PIVC fragments, since they are designed to be radiopaque [11], a CT scan is also capable of accurately localizing foreign material and aiding in the pre-operative surgical planning and approach, particularly in cases where the cut end has migrated into the chest cavity. In one study, a comparison of the agreement between CT reports and surgical findings in dogs and cats undergoing thoracic procedures was conducted. These pathological findings were most common in lung masses or pleural effusion. Results showed an 86% agreement rate and a sensitivity of 93%, demonstrating the clinical utility of CT in preoperative assessment of thoracic surgical cases [16]. Our case report supports the diagnostic utility of CT scans for foreign material identification. Accurately localizing the foreign body is important for preventing unnecessary surgical dissection and successful retrieval of the foreign body [17].

While lateral thoracotomies are a common surgical procedure in veterinary medicine for a variety of purposes, foreign material migration into the pulmonary parenchyma is not a common indication for performing this type of procedure. One retrospective study evaluated reasons for performing a lateral thoracotomy, and foreign material retrieval into the pulmonary parenchyma was not listed [18]. The success of thoracotomy in this case may support prompt surgical intervention for the removal of foreign bodies in the pulmonary parenchyma. Some studies have reported that failure to remove migrating foreign bodies in the lung parenchyma can lead to clinical complications such as lobar consolidation, pneumothorax, pyothorax, or abscessation [19,20]. Although these foreign materials were more commonly grass awns, they still have the potential to cause an embolism or infectious processes. While intrathoracic migrating foreign bodies are uncommon in small animals, prompt removal of a migrating foreign body in the lungs is important to avoid future complications.

A literature review comparing PIVC foreign bodies in humans and those in animals reveals differences between diagnosis and management. In human medicine, IVC embolism is more often incidentally found months to years later with patients often being asymptomatic. Common causes of PIVC damage are catheter explantation and exchange, as explained by Surov et al. However, if the foreign material is left in situ, complications have been reported in human medicine that include arrhythmias, dyspnea, cough, thoracic pain, and in rare cases sepsis. One study indicated a mortality rate of 1.8% in 215 reported cases [9]. According to Surov et al., these complications and the mortality rate are sufficient to justify surgical extraction in human medicine. In veterinary medicine, identification of a PIVC foreign body is likely immediate via observation after the IVC has been removed from the patient. A potential complication rate of PIVC embolism has not been established in veterinary medicine due to the limited number of reports published [7]. Keen observation after PIVC removal is important for quickly detecting if portions of the PIVC have dislodged and to provide early intervention. To avoid the severance of the catheter tip when removing the intravenous catheter, the bandage scissors should always be held parallel to the long axis of the forearm and away from the catheter hub. Additionally, sliding the catheter out in a straight manner upon removal to avoid the potential breakage of the catheter tip at the hub level is recommended. Providing adequate restraint during the maneuver is also important to reduce patient self-trauma while removing the IV catheter.

## 4. Conclusions

Prompt identification of PIVC embolism is important for early intervention and PIVC foreign body removal. If survey radiographs do not reveal the location of the foreign material, a full-body CT should be considered for the localization of the PIVC embolism to confirm diagnosis and for pre-operative planning if surgical intervention is indicated. A thoracotomy with PIVC removal can be considered for the removal of foreign material from the pulmonary parenchyma, depending on the patient’s health status, to avoid future complications.

## Data Availability

The original contributions presented in this study are included in the article. Further inquiries can be directed to the corresponding author.

## Abbreviations

The following abbreviations are used in this manuscript:

PIVC	Peripheral intravenous catheter
CT	Computed tomography
HU	Hounsfield units
CBC	Complete blood count
NSAID	Non-steroidal anti-inflammatory drug
PTT	Partial thromboplastin time
